# Gastrin, Cholecystokinin, Signaling, and Biological Activities in Cellular Processes

**DOI:** 10.3389/fendo.2020.00112

**Published:** 2020-03-06

**Authors:** Qiang Zeng, Lei Ou, Wei Wang, Dong-Yu Guo

**Affiliations:** ^1^Health Management Institute, People's Liberation Army General Hospital, Beijing, China; ^2^Department of Clinical Laboratory, Xiamen Huli Guoyu Clinic, Co., Ltd., Xiamen, China

**Keywords:** gastrin, CCK, G protein-coupled receptor, cancer, imaging, therapeutics

## Abstract

The structurally-related peptides, gastrin and cholecystokinin (CCK), were originally discovered as humoral stimulants of gastric acid secretion and pancreatic enzyme release, respectively. With the aid of methodological advances in biochemistry, immunochemistry, and molecular biology in the past several decades, our concept of gastrin and CCK as simple gastrointestinal hormones has changed considerably. Extensive *in vitro* and *in vivo* studies have shown that gastrin and CCK play important roles in several cellular processes including maintenance of gastric mucosa and pancreatic islet integrity, neurogenesis, and neoplastic transformation. Indeed, gastrin and CCK, as well as their receptors, are expressed in a variety of tumor cell lines, animal models, and human samples, and might contribute to certain carcinogenesis. In this review, we will briefly introduce the gastrin and CCK system and highlight the effects of gastrin and CCK in the regulation of cell proliferation and apoptosis in both normal and abnormal conditions. The potential imaging and therapeutic use of these peptides and their derivatives are also summarized.

## Introduction

Multicellular organisms have developed a delicate and efficient cellular regulatory system to maintain a balanced cell proliferation, differentiation, and apoptosis. Aberrant cellular regulation usually results in pathophysiology, for instance, carcinogenesis resulting from infinite cell proliferation, escape from apoptosis, and metastatic invasion. The gut is the largest endocrine organ in the body that expresses over 30 gut hormone genes and a wealth of bioactive peptides (1). As two of the first gastrointestinal peptides discovered, gastrin and CCK are known to play important roles in digestive processes including gastric acid secretion, pancreatic enzyme release, gallbladder emptying, gut motility, and energy homeostasis (2–4).

However, accumulating *in vitro* and *in vivo* studies have demonstrated that gastrin and CCK integrate and coordinate a rich network of information exchange pathways in cellular processes of proliferation and apoptosis, and under certain circumstances, they contribute to the pathogenesis and progression of some types of tumors (5). Indeed, gastrin, CCK and their cognate receptors (CCK2R and CCK1R, discussed in detail in section The Receptors for Gastrin and CCK) have been reported to be expressed and involved in several adenocarcinomas originated in stomach, colon, pancreas, esophagus, and gallbladder, as well as some tumors in the brain (6, 7). However, the gastrin and CCK regulatory systems are complex and intricate due to wide distribution of the hormone genes, cell-specific alternative splicing and post-translational modifications, and activation of multiple signal transduction pathways. In this review, we aim to briefly introduce the gastrin and CCK systems regarding peptide biosynthesis, cellular expression, receptor activation, downstream signaling pathways, and involvement in proliferative and apoptotic responses in normal and malignant conditions. The underlying mechanisms attributed to the peptide-induced cellular effects and potential imaging and therapeutic applications will be elaborated.

## The Gastrin and CCK System

### Gastrin and CCK

As two of the first gastrointestinal hormones identified, the gastrin and CCK were originally discovered as hormonal regulators in stomach and small intestine in 1906 and 1928, respectively (8, 9). Subsequent isolation and structure determination of gastrin and CCK in the 1960s (10, 11) attracted researchers to investigate the biology, physiology, and pharmacology of these two peptides. In humans, the genes encoding gastrin and CCK precursors are located on chromosome 17q21 and 3p22-p21.3, respectively (12, 13). Both gastrin and CCK exist in multiple molecular forms following cell-specific post-translational processing. Among these peptides, the biologically active gastrin includes progastrin, Gly-extended gastrin-17 or −34 (Ggly), and the amidated gastrin-17 or −34 (Gamide), whereas the biologically active CCKs include Gly-extended and amidated CCK-33, -58, -22, and -8 (4, 14). In humans, amidated G-17 and CCK-33 predominate in plasma, and amidated CCK-8 predominates in the brain (15, 16). It should be noted that all bioactive gastrin and CCK peptides share the same amidated COOH-terminal pentapeptide (Gly-Trp-Met-Asp-Phe-NH2) motif, which is exceedingly well-conserved during evolution and comprises the minimal sequence (pharmacophore) required for biological activity and receptor activation (17).

In response to food intake, gastrin is synthesized and released by mucosal G cells to stimulate enterochromaffin-like (ECL) cells to secrete histamine, which further induces acid release from parietal cells through activation of H_2_ histamine receptors, whereas CCK is predominantly produced and secreted by upper small intestinal I cells to stimulate gallbladder contraction and pancreatic enzyme secretion. As classical gut hormones and potent neurotransmitters, gastrin and CCK are widely distributed in gastrointestinal tract, CNS, and peripheral neurons (4, 18). However, CCK has also been suggested to stimulate spermatozoan fertilization, exert anti-inflammatory effects, promote sodium excretion into the urine, and predict the risk of mortality in heart failure, consistent with its expression in male germ cells (19), cells of immune system (20–23), renal cortex and medulla (24), and cardiomyocytes (25), respectively.

In addition to the acute digestive effects, both gastrin and CCK have been suggested to exert potent proliferative and anti-apoptotic effects by contributing to pathogenesis and progression of cancer (26, 27). Indeed, hypergastrinemia induced by proton pump inhibitor (PPI) together with *Helicobacter pylori* (*H. pylori*) infection correlates with higher risk of gastric and colorectal cancer in experimental animal models (28). Increased expression of gastrin and/or CCK were observed in human gastric adenocarcinoma (29, 30), colorectal carcinoma (31, 32), and pancreatic cancer (33), over that of the corresponding normal tissues, indicating potential roles of these peptides in promoting carcinogenesis (6).

### The Receptors for Gastrin and CCK

The broad range of physiological functions of gastrin and CCK are mediated by two cognate receptors, which belong to rhodopsin-like G protein-coupled receptors characterized with the hallmark structure of seven transmembrane domains and the alternating NH_2_ terminus and COOH tail. Gastrin receptor is also referred as CCK2 receptor (CCK2R, previously also called CCK-B receptor) based on the facts that (1) gastrin and CCK share the core sequence required for biological activities of both peptides and activation of the receptor; (2) gastrin and CCK have comparable affinity and potency for CCK2R; (3) CCK2R mediates almost all the classical physiological activities of gastrin (34, 35). However, CCK1 receptor (CCK1R, previously also called CCK-A receptor) binds and responds to CCK with a 500- to 1,000-fold higher affinity and potency than gastrin (36). Therefore, CCK1R is considered as the cognate receptor for CCK (35).

Using primarily cultured mucosal cells, Sachs and colleagues showed that both gastrin and CCK exert essentially equal potency in stimulating acid secretion from gastric parietal cells and histamine release from mucosal cells (37, 38), which are in line with their comparable affinity and potency for CCK2R. However, non-sulfated CCK-8 is less potent and less effective than gastrin in inducing DNA synthesis of isolated ECL cells, as assessed by 5-bromo-2′-deoxyuridine incorporation and ELISA (38). In addition, infusion of gastrin or CCK-8 in rats stimulates histamine synthesis and ECL hyperplasia except that CCK-8 fails to induce ECL hyperplasia even the circulating CCK-8 levels are 10-fold above normal (39, 40). The differential growth responses between gastrin and non-sulfated CCK-8 might be due to lower stability of non-sulfated CCK-8 caused by endopeptidases from the cell culture and/or different binding states of CCK2R are existing for gastrin and non-sulfated CCK-8 (38). Additional studies employing molecular cloning and radioligand binding might provide further explanations.

Table 1 summarizes the general distribution of CCK2R and CCK1R in healthy adult mammals. CCK2R is predominantly expressed in the brain and selected regions in the gastrointestinal tract, including gastric epithelial parietal cells, ECL cells, and D cells, pancreatic acinar cells, myenteric neurons, monocytes and T lymphocytes, and human peripheral blood mononuclear cells (18, 41, 42, 47, 48, 56, 57), whereas CCK1R is mainly found in pancreatic acinar cells, gallbladder smooth muscles, gastric mucosal chief and D cells, as well as cerebral and peripheral neurons (6, 46, 50–52).

**Table 1:** Expression of CCK2R and CCK1R in normal tissues.

**Receptor**	**Expression sites**	**Techniques**	**Species**	**References**
CCK2R	Present in gastric mucosal parietal cells, enterochromaffin-like, and D cells.	Northern blotting, IHC, and RT-PCR	Canine, guinea pig, and/or human	(41–44)
	Present in pancreatic cells.	Autoradiography	Human	(45)
	Present in cortex, olfactory regions, hippocampal formation, septum, and interpeduncular nucleus, and amygdaloid nuclei.	*In situ* hybridization	Rat	(46)
	Present in duodenum myenteric neurons.	Autoradiography	Canine	(42)
	Present in cells of the immune system including leukemia cell lines derived from myeloid, T- and B- lymphoid, and peripheral blood mononuclear cells.	RT-PCR	Human	(47, 48)
	Present in rat brain and the fundus mucosa but absent in the rest of the digestive tract, pancreas, pancreatic islets, or kidney.	Northern blotting	Rat	(49)
	Present in rat brain and in the mucosa from the fundus and antrum but totally absent in the intestines, pancreas, pancreatic islets, and kidney Present in human brain, stomach, and pancreas but absent in the kidney.	RT-PCR and Southern blotting	Rat and human	(49)
CCK1R	Present in pancreatic acinar cells.	Autoradiography	Guinea pig	(50)
	Present in gallbladder smooth muscles.	Autoradiography	Bovine and human	(45, 51)
	Present in chief and D cells of gastric mucosa and absent in fundic mucosal histamine-containing cells.	Radioligand binding assay	Canine and guinea pig	(52, 53)
	Present in cortex, olfactory regions, hippocampal formation, septum, and interpeduncular nucleus, as well as hypothalamic nuclei including paraventricular nucleus, arcuate nucleus, and medial preoptic area.	*In situ* hybridization	Rat	(46)
	Present in fundus mucosa and pancreas but absent in the remaining GI tract or brain.	Northern blotting	Rat	(49)
	Present in rat brain and the mucosa of the fundus, antrum, duodenum, and colon, kidney, pancreas and pancreatic islets but absent in the lieum; Present in human brain, stomach, pancreas, and kidney.	RT-PCR and Southern blotting	Rat and human	(49)
	Present in gallbladder, intestine, brain, ovary, spleen, thymus, and ductal cells.	RT-PCR and *In situ* hybridization	Human	(54)
	Present in brain capillary endothelial cells.	IHC and Western blotting	Rat	(55)

*IHC, immunohistochemistry; RT-PCR, reverse transcriptase polymerase chain reaction*.

Table 2 summarizes the localization and actions of gastrin, CCK, and their receptors on different types of human cancers (indicated as percentage of positive tissues expressing the corresponding ligand or receptor). A variety of human adenocarcinomas in the stomach, pancreas, colon, rectum, esophagus, lung, liver, medullary thyroid, overexpress CCK2R and/or CCK1R than the matched normal tissues (61, 62, 71, 79, 81, 82), serving as the basis for CCK2R- or CCK1R-targeted tumor imaging and therapy (84). Indeed, intensive chemical, physiological, and pharmacological studies provide novel diagnostic and therapeutic agents for the tumors overexpressing CCK2R and/or CCK1R (85).

**Table 2:** Expression and function profiles of gastrin, CCK, and their receptors in human cancer cell lines and tissues.

**Tumor types**	**Percentages of positive expression (positive/total)**				**Techniques**	**Effects on tumor cells**	**References**
	**Gastrins^a^**	**CCK**	**CCK1R**	**CCK2R**			
**Gastric cancer**							
Tumor cell lines	Present in MKN45G and SGC-7901	NA^b^	NA^b^	Present in ECC10, SGC-7901, TMK-1, and HSC-39; Absent in AGS, ECC12, MKN-1, HGC27, HSK-TC, GCIY, KATOIII, OKAJIMA	FC, IHC, and/or Northern blotting	The gastrin-CCK2R system plays an important role in the elevated morphology of gastric tumors.	(29, 58–60)
Tumor tissues	36% (8/22)	NA^b^	NA^b^	NA^b^	FC and IHC	Treatment of anti-gastrin-17 antiserum significantly reduces proliferation of gastric tumor cells.	(58)
	0 (0/14)	4/14 (29%)	5/14 (36%)	1/14 (7%)	RT-PCR	CCK and CCK1R might play a more important role than for gastrin and CCK2R in gastric cancers.	(61)
	NA^b^	NA^b^	63% (5/8)	88% (7/8)	RT-PCR	Local or systemic originated-CCK might influence the growth of esophageal tumors.	(62)
	100% (15/15)	NA^b^	NA^b^	100% (15/15)	IHC	The expression levels of progastrin, Ggly, Gamide, and CCK2R positively correlates with the degree of gastric lesions.	(30)
	73% (22/30)	NA^b^	NA^b^	100% (30/30)	RT-PCR	Co-expression of gastrin and CCK2R might contribute to progression of gastric cancer.	(29)
	48% (133/279)	NA^b^	NA^b^	57% (158/279)	IHC	Gastric carcinoma tissues expressing both gastrin and CCK2R have a poorer prognosis than those negative for both.	(63)
	NA^b^	NA^b^	NA^b^	65% (31/48)	IHC	The gastrin system plays an important role in the elevated morphology of gastric tumors.	(60)
	NA^b^	NA^b^	NA^b^	0 (0/10)	Northern blotting	CCK2R might not be involved in gastric tumor.	(59)
	NA^b^	NA^b^	0 (0/27)	7% (2/27)	Autoradiography	CCK2R and CCK1R might not be involved in gastric tumor.	(64)
**Pancreatic cancer**							
Tumor cell lines	Present in PANC-1, BxPC-3, AsPC-1, Capan-1, and MIA PaCa-2	Present in PANC-1, BxPC-3, and AsPC-1Absent in MIA PaCa-2	Present in PANC-1, Capan-1; Absent in BxPC-3, AsPC-1, and MIA PaCa-2	Present in PANC-1, BxPC-3, AsPC-1, Capan-1, and MIA PaCa-2	Radioligand binding and real time-PCR	The autocrine production of gastrin and CCK are important for stimulating pancreatic tumor cell growth.	(65–69)
Tumor tissues	NA^b^	NA^b^	100% (22/22)	100% (22/22)	RT-PCR and *in situ* hybridization	CCK1R might serve as selective bio-marker for pancreatic adenocarcinoma.	(54)
	NA^b^	NA^b^	90% (27/30)	NA^b^	RT-PCR and *in situ* hybridization	Increased expression of CCK1R might promote pancreatic malignancies.	(70)
	Up to 91%	NA^b^	NA^b^	95% (21/22)	IHC	CCK2R, progastrin, Ggly, and Gamide might promote pancreatic malignancy in an autocrine manner.	(33)
	Up to 74% (14/19)	0 (0/18)	67% (12/18)	100% (18/18)	RIA and RT-PCR	A local regulatory mechanism through gastrin and CCK2R, but no CCK mechanism, might be involved in pancreatic carcinoma.	(71)
	NA^b^	NA^b^	0 (0/32)	9% (3/32)	Autoradiography	Ductal pancreatic tumor cells very rarely express CCK1R and CCK2R.	(72)
**Colorectal cancer**							
Tumor cell lines	Present in LoVo, HCT-15, HT-29, Caco2, SkCo15 Absent in COLO−201, DLD1, SW403	NA^b^	NA^b^	Present in Caco2, Sk-Co15, HT-29.18 glu, and HT-29.18 gal.	Northern blotting and RT-PCR	Incomplete processing and low level of expression of gastrin were observed in five human colon carcinoma cells.	(32, 59, 73)
Tumor tissues	NA^b^	NA^b^	NA^b^	67% (45/67)	Radioligand binding assay	CCK2R content of colon cancers may have prognostic and therapeutic significances.	(74)
	21% (6/28)	NA^b^	NA^b^	NA^b^	FC and IHC	Gastrin but not CCK promotes growth of human gastric adenocarcinoma cells.	(58)
	100% (15/15)	NA^b^	NA^b^	NA^b^	RIA	Gastrin precursors are more abundant than amidated-G in neoplastic colon.	(75)
	Up to 97% (22/23)	NA^b^	NA^b^	NA^b^	Ribonuclease protection, IHC, Southern blotting, and RT-PCR	About 97 and 87% of colorectal adenocarcinomas express Gamide and progastrin, respectively, which might promote proliferation of colorectal tumor.	(76)
	Up to 100% (44/44)	NA^b^	NA^b^	NA^b^	RIA	Expression of progastrin and Ggly is increased in tumor tissues than controls.	(31)
	Up to 100% (12/12)	NA^b^	NA^b^	NA^b^	Northern blotting and RT-PCR	Solid colonic tumors contain higher levels of progastrin than normal colonic tissues.	(32)
	NA^b^	NA^b^	NA^b^	20% (2/10)	Northern blotting	Indicates a role of CCK2R in growth and differentiation of colorectal carcinomas.	(59)
	86% (96/112)	NA^b^	NA^b^	11% (13/112)	RNase protection assay, radioligand binding	The gastrin system exists in an autocrine proliferative loop in colorectal tumor.	(77)
	87% (26/30)	NA^b^	NA^b^	77% (23/30)	RT-nested PCR, Southern blotting	Gastrin might stimulate the growth of human tumor cells likely through a receptor other than CCK1R and CCK2R.	(73)
	NA^b^	NA^b^	42% (5/12)	17% (2/12)	RT-PCR	Local or systemic originated-CCK might influence the growth of colorectal tumor.	(62)
	NA^b^	NA^b^	0 (0/25)	4% (1/25)	Autoradiography	CCK2R and CCK1R might not be involved in colorectal tumor.	(64)
	44% (35/79)	NA^b^	NA^b^	38% (30/79)	IHC, RT-PCR	Co-expression of gastrin and CCK2R message is significantly increased in colorectal tumor.	(78)
**Esophageal cancer**							
Tumor tissues	NA^b^	NA^b^	63% (5/8)	0/8	RT-PCR	Local or systemic originated-CCK might influence growth of esophageal tumor.	(62)
	NA^b^	NA^b^	NA^b^	58.3% (7/12)	RT-CPR, Northern blotting	Gastrin-induced signaling through CCK2R promotes tumor cell proliferation.	(79)
	100% (4/4)	NA^b^	NA^b^	75% (3/4)	RT-PCR	Almost all esophageal tumors express gastrin and CCK2R.	(80)
**Other types of cancer**							
Small cell lung cancer cell lines	NA^b^	NA^b^	NA^b^	Present in H60, Lu134A, and Lu139; absent in PC6, Lu134B, Lu135, and PC14	Northern blotting	The majority of human small cell lung cancer cells express CCK2R.	(59)
Small cell lung cancer tissues	NA^b^	NA^b^	NA^b^	100% (10/10)	RT-PCR	The CCK2R might be a good prognostic and therapeutic target for small cell lung cancer.	(81)
Thyroid cancer tissues	NA^b^	NA^b^	8%(2/23)	92% (21/23)	Autoradiography	CCK2R might be utilized as diagnostic and therapeutic target for thyroid cancer.	(82)
Hepatic metastasis	71% (5/7)	NA^b^	NA^b^	100% (7/7)	RT-nested PCR, Southern blotting	A novel receptor different from CCK1R and CCK2R might be involved in gastrin-induced proliferative effects on hepatic tumor.	(73)
Gallbladder tumor tissues	NA^b^	NA^b^	77% (72/94)	NA^b^	IHC and IMB	CCK1R expression is significantly increased in gallbladder cancer and associated with the degree of tumor differentiation.	(83)

a *Includes three forms of gastrins: progastrin, Ggly, and Gamide*.

b *Not assessed*.

*FC, flow cytometry; RIA, radioimmunoassay; RT-PCR, reverse transcriptase polymerase chain reaction; IHB, immunoblotting; IHC, immunohistochemistry; Gamide, amidated gastrin; Ggly, Gly-extended gastrin*.

Another line of evidence, the identification and characterization of the CCK2R splice variants and mutations, further confirmed the involvement of the gastrin and CCK system in cell proliferation and cancer pathogenesis. Indeed, CCK2Ri4sv, an CCK2R splice variant containing intron 4 and therefore additional 69 amino acids in the third intracellular loop, was identified in patients with colorectal cancer (86). Further *in vitro* functional studies showed that CCK2Ri4sv exhibits constitutive activation of signaling pathways resulting in enhanced Ca^2+^ levels and cell proliferation in both primary and Balb3T3 human colorectal tumor cells (86). In contrast, *in vitro* expression of CCK2Ri4sv in human epithelial HEK293 cells does not affect cell growth (87). Interestingly, compared to CCK2R-HEK293-xenografted mice, the CCK2Ri4sv-HEK293-xenografted mice have significantly increased tumor growth, which is associated with a constitutive, Src-dependent increase in the transcription factor hypoxia-inducible factor-1α and secretion of vascular endothelial growth factor (87). Other naturally occurring mutations in the *CCK2R* gene, such as V287F and R396C, were also shown to promote cell proliferation or angiogenesis through increase in Src-dependent secretion of cytokines (88).

Several cellular models have been utilized to investigate the cellular effects of gastrin and CCK, including (1) cells endogenously expressing CCK2R and CCK1R including human pancreatic tumor PANC-1 and Capan-1 cells (65–67), rat brain E18 neuroblasts (89); (2) cells endogenously expressing only CCK2R, such as rat pancreatic tumor AR42J cells (90), human pancreatic tumor BxPC-3, MIAPaCa-2, and AsPC-1 cells (67, 68, 91), rat pituitary adenoma GH3 cells (92), and human colon cancer HT-29 cells (93); (3) cells ready to be transiently or stably transfected with CCK2R or CCK1R, such as human gastric adenocarcinoma AGS and MKN-45 cells (94, 95), rat intestinal epithelial RIE-1 cells (96), Rat-1 and mouse Swiss 3T3 (also named NIH3T3) fibroblasts (97–99), Chinese hamster ovary CHO cells (99).

### Intracellular Signaling Pathways

**Figure 1:**
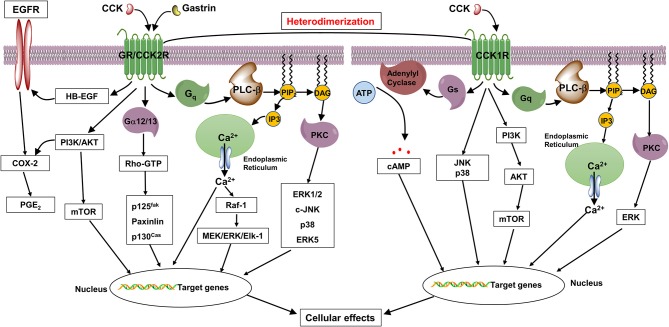
Proposed diagrams of gastrin- and CCK-induced signaling pathways through CCK2R and CCK1R in normal and tumor cells. In response to gastrin and CCK, CCK2R couples to Gq and Gα_12/13_ proteins to promote cell proliferation and inhibit apoptosis through activation of PLC/Ca^2+^/PKC, MAPK, p125fak, Src, and PI3K/AKT cascades, as well as transactivation of EGFR, whereas CCK1R couples to Gq and Gs to exhibit trophic effects through activation of PLC/Ca^2+^/PKC, AC/cAMP/PKA, MAPK, and PI3K/AKT pathways.

Once activated by the ligands, the CCK2R and CCK1R located on the cell surface undergo conformational changes and trigger a complex intracellular network of signaling pathways. As shown in Figure 1, both CCK2R and CCK1R can signal through Gq protein to activate phospholipase Cβ (PLCβ), resulting in hydrolysis of phosphatidylinositol 4,5-bisphosphate into inositol trisphosphate and diacylglycerol. Furthermore, second messenger diacylglycerol, together with inositol trisphosphate-induced Ca^2+^ efflux from endoplasmic reticulum, stimulate the phosphorylation of protein kinase C (PKC) isoforms to activate downstream effector proteins such as mitogen-activated protein kinases (MAPKs, important regulators in cell proliferation, differentiation, survival, and apoptosis) and inflammatory regulator NF-κB (35, 100).

In addition, both receptors are able to induce PKC-independent activation of MAPK and PI3K/AKT/mTOR signaling pathways (35, 101). A variety of non-receptor tyrosine kinases, including protooncogene Src kinase, focal adhesion tyrosine kinase (FAK, involved in cell morphology, cell motility, and invasion), and Janus kinase (JAK, involved in cell proliferation, differentiation, apoptosis, and oncogenesis) have also been reported to be activated by CCK2R and/or CCK1R (97, 102, 103). Furthermore, likely through activation of heparin-binding epidermal growth factor (EGF)-like growth factor, CCK2R activation was also reported to lead to transactivation of the EGF receptor (EGFR), a transmembrane tyrosine kinase receptor that plays important roles in cell growth, apoptosis, and migration (96, 104).

However, only CCK1R can couple to Gs protein to activate adenylyl cyclase, a cell membrane enzyme that catalyzes the cytoplasmic ATP into cAMP. The intracellular cAMP acts as second messenger to activate protein kinase A, which further stimulates the phosphorylation of cAMP response element-binding protein, a well-known transcription factor that affects a wealth of downstream genes (105, 106). In addition, the activation of nitric oxide (NO)/cGMP signaling cascade have been reported to be mediated by CCK1R in CHO cells and rodent pancreatic acini cells expressing CCK1R (107, 108).

## Gastrin and CCK in Cell Proliferation

### Gastrin and CCK Effects *in vitro*

A number of *in vitro* studies showed that gastrin and/or CCK induce a significant but modest increase in DNA synthesis in CCK2R-expressing cells, about 1.5-fold in AR42J cells, 1.6-fold in GH3 cells, up to 1.6-fold in HT-29 cells, 1.8-fold in CHO cells, 3-fold in Rat-1 cells, and 4-fold in Swiss 3T3 cells over the corresponding unstimulated control cells (98, 99, 109–111). Although gastrin and CCK exert similar growth-promoting effect in different cell models, the mechanisms attributed for the ligand-stimulated trophic effects seem to be cell specific. In AR42J cells, gastrin was shown to stimulate cell proliferation through MEK/ERK2/Elk-1-induced upregulation of c-*fos* gene, an early response gene associated with cell growth (94, 109). This activation is PKC-dependent and requires the small GTP-binding RhoA, the CA rich G sequence of the SRE promoter, and transcription factors Elk-1 and Sap-1a which bind to the E26 transformation specific motif (112). Interestingly, in GH3 cells with a similar CCK2R expression and gastrin/CCK2R binding kinetics compared to AR42J cells, the same group further showed that gastrin promotes cell growth in Ca^2+^-dependent manner, without activation of ERK1/2 (110).

In addition, gastrin and CCK stimulate cell proliferation in ERK1/2- and p74raf-1 kinase-dependent but Gi-independent manner in CCK2R-transfected Rat-1 cells (98), whereas it involves PKC/Ca^2+^- and Src-dependent activation of p38 pathway in CHO-CCK2R cells (103). In CCK2R-expressing Swiss 3T3 cells, gastrin induces G_1_/S cell cycle transition and cell proliferation through upregulation of cyclin D1, D3, and E, activation of cyclin-dependent kinases, and hyperphosphorylation of retinoblastoma protein (113). Gastrin-induced cyclin D1 transcription activity is mediated through activation of β-catenin and CREB pathways in AGS-CCK2R cells (114).

Cyclooxygenase-2 (COX-2), an inflammatory regulator critical for prostaglandin synthesis, have been suggested to be a downstream player of CCK2R. Indeed, gastrin increases transcriptional level of COX-2 in several cell lines, although the underlying mechanisms are different. In RIE-1 cells, the mRNA level of COX-2 is increased through activation of ERK5 and transactivation of the EGFR (96), whereas in Swiss 3T3 cells, it does not require PKC activity, activation of ERK1/2, or transactivation of EGFR (115). Gastrin-dependent COX-2 expression is inhibited by pretreatment with CCK2R antagonist L365,260, but not by pretreatment with CCK1R antagonist L364,714, indicating CCK2R but not CCK1R mediates gastrin-induced upregulation of COX-2 (115). In HT-29 cells, gastrin stimulates COX-2 expression via ERK1/2 and PI3K/AKT pathways (93). Together with the evidence that the COX-2 inhibitor L-745,337 reverses gastrin-induced DNA synthesis and cell growth (93), it is reasonable to conclude that COX-2 is responsible for the trophic effects of gastrin.

As the most abundant peptide neurotransmitter in the brain, the effects of gastrin and CCK on neuronal proliferation have been also investigated. In rat brain E18 neuroblasts, the group of Bragado showed that CCK promotes neuroblast proliferation by inducing tyrosine phosphorylation of adaptor proteins p130^Cas^ and paxillin (89), two key components of focal adhesion complexes (116, 117), and phosphorylation of PKB/AKT and ERK1/2, followed by stimulation of DNA-binding activity of AP-1 (89). Furthermore, pharmacological blockade of CCK2R signaling with a potent and selective non-peptide CCK2R antagonist CR2945 inhibits CCK-stimulated ERK1/2 phosphorylation by over 50%, whereas totally antagonizes gastrin-stimulated ERK1/2 phosphorylation, indicating the proliferation-promoting effects of CCK on rat brain neuroblasts are mediated by both CCK1R and CCK2R (89).

### Gastrin and CCK Effects *in vivo*

The trophic actions of gastrin on gastric mucosa were demonstrated by a sequence of *in vivo* studies. Two early clinical studies reported that inhibition of gastrin synthesis by gastrectomy results in atrophy of the residual mucosa, suggesting the involvement of gastrin in the regulation of mucosal cell growth (118, 119). Indeed, continuous administration of pentagastrin to male Wistar rats results in an increase in both density and population of gastric mucosal parietal cells (120, 121). Inspired by the above discoveries, Willems and colleagues, using histamine immunocytochemistry and autoradiography after labeling of mucosal specimens with ^3^H-thymidine, first showed sustained administration of porcine gastrin causes a marked increase in DNA synthesis and in the mitotic index in canine fundic mucosa (122). Similarly, endogenous and exogenous hypergastrinemia, induced by antrocolic transposition, antral exclusion, and subcutaneous infusion of G17 were reported to lead to elevated cell proliferation rates of mucosal cells and oxyntic mucosal thickness in rats (123–125). Using BxPC-3 cell-xenografted mice, Smith et al. first demonstrated that gastrin, but not CCK, stimulates pancreatic tumor growth in a tonic and autocrine fashion (67, 68).

Extensive rodent studies have demonstrated that hypergastrinemia induced by continuous treatment of acid-suppressing drugs, including PPIs and H_2_ histamine receptor antagonists, results in mucosal hyperplasia and ECL cell hyperplasia. In rare cases, ECL cell neuroendocrine tumors (NETs) might develop although these generally remain benign (126, 127). However, *Mastomys*, a hypergastrinemic rodent model that is genetically susceptible to spontaneous formation of gastric NETs, can develop gastric carcinoid tumor in the presence of normal serum gastrin levels, likely through the constitutive activation of CCK2R (128, 129). In humans, gastrin is known to promote proliferation of ECL cells and pathogenesis of gastric NETs. Indeed, hyperplasia-dysplasia-neoplasia processes in ECL cell populations were reported in hypergastrinemic conditions (130–132). However, it is still of debate whether hypergastrinemia alone is sufficient to result in ECL cell NETs. Patients with sporadic Zollinger-Ellison syndrome (ZES) rarely develop gastric NETs even though their circulating gastrin levels are over 10-fold above normal for a long period of time (133, 134). In contrast, gastric NETs were reported in 13–30% of patients combined with ZES and familial multiple endocrine neoplasia type I (MEN-1, an autosomal dominantly inherited disorder caused by inactivation of MEN-1 gene), indicating that gastrin and genetic factors are both important for the formation of gastric NETs (135, 136).

In addition, the ability of CCK to induce pancreatic hyperplasia and hypertrophy was also reported by several *in vivo* studies. Sustained subcutaneous injection of cerulein, a structural and functional homolog of CCK, in rats initiates a significant dose- and time-dependent increase in pancreatic weight and DNA, RNA, and protein contents (137, 138). Similar effects of CCK-8 were observed in male Wistar rats by Rosewicz et al., who further reported the trophic actions of CCK analog might be mediated by induction of pancreatic ornithine decarboxylase (ODC) activity and subsequent accumulation of polyamines, which are closely involved in cellular growth and proliferation due to their ability to facilitate almost all aspects of DNA, RNA, and protein syntheses (139, 140). Indeed, the cerulein-induced pancreatic acinar cell growth is inhibited by an irreversible ODC inhibitor α-difluoromethylornithine and this inhibition is further reversed by administration of the small polyamine putrescine (141, 142).

Very recently, an elegant study by Stanic and his colleagues revealed that CCK, signaling through CCK1R, is involved in regulating neurogenesis in the female adult mouse brain (143). In this study, transgenic mice lacking *Cck1r* (*Cck1r*^−/−^) have decreased proliferating cells in the subgranular zone of the dentate gyrus and rostral migratory stream, with 42% and 29% lower number of cells immune-stained with Ki67 (a nuclear protein associated with cell proliferation) and Doublecortin (a microtubule-associated protein expressed in migrating and differentiating neurons), respectively (143–145). The decreased proliferating precursor cells in female *Cck1r* ^−/−^ mice further result in fewer migratory neuroblasts and tyrosine hydroxylase-immunoreactive mature interneurons in the olfactory bulb compared to the wild-type (WT) mice (143). Similarly, the proliferation-promoting effects of CCK on neurons were confirmed by Reisi et al., who showed that intraperitoneal injection of CCK in male Wistar rats promotes neurogenesis, as evidenced by significantly enhanced Ki-67 positive cells in the granular layer of hippocampal dentate gyrus than those treated with placebo (146).

### Mechanism of Proliferative Actions of Gastrin and CCK

Physiologically, two sources of gastrin, originating from mucosal G cells and gastrointestinal tumors, have been suggested to act in different fashions to promote the pathogenesis and development of certain types of cancer. However, the signaling pathways mediating gastrin- and CCK-induced growth-promoting effects are still not fully understood. Since the processes of cellular proliferation are regulated by a complex signaling network of phosphorylation events initiated by the interaction of growth factors with their specific cellular receptors (147), we depicted the critical pathways that might be responsible for gastrin- or CCK-induced trophic effects in Figure 1, based on results obtained from various cell models. These include Gq-mediated phosphatidyl inositol turnover, Ca^2+^ mobilization, and PKC phosphorylation, Gs-mediated intracellular cAMP accumulation, Gα_12/13_-mediated Rho-dependent tyrosine phosphorylation of FAKs, activation of MAPK, Src, and PI3K/AKT, as well as transactivation of EGFR, which ultimately results in the regulation of target genes and contribute to proliferative actions of gastrin or CCK (5, 85, 148).

Interestingly, heterodimerization of CCK2R and CCK1R has been also implicated in promoting CCK-induced cell proliferation. In CCK1R-tansfected COS cells, the presence of CCK1R oligomeric complexes was demonstrated by Cheng and Miller in bioluminescence resonance energy transfer and co-immunoprecipitation experiments (149). CCK occupation of CCK1R induces the dissociation of those complexes in a concentration-dependent but phosphorylation-independent manner (149). The same group further showed that heterodimers composed of CCK2R and CCK1R display novel functional and regulatory properties with increased intracellular Ca^2+^ mobilization and delayed receptor internalization in response to CCK stimulation compared to these in cells only expressing individual receptor (150). It should be noted that CCK1R/CCK2R heterodimer-expressing COS cells tend to have enhanced CCK-induced cell proliferation responses, indicating a stimulant role of heterodimerization on the trophic effects of CCK (150).

## Gastrin and CCK in Apoptosis

### Gastrin and CCK Effects *in vitro*

Compared to the well-described proliferative actions of gastrin and CCK, their involvement in the regulation of apoptosis is, however, poorly understood. To date, whether gastrin and CCK exert apoptotic or anti-apoptotic effect is still of debate and might be dependent on the exact physiological and pathological conditions.

Using flow cytometry and terminal deoxynucleotidyl transferase-mediated dUTP-FITC nick end labeling (TUNEL) method, Todisco et al. first demonstrated that gastrin reverses serum withdrawal-induced cellular apoptosis and promotes AR42J cell survival through PI3K- and p38-dependent activation of AKT (151). The same group further showed AKT inhibits apoptosis through activation of the pro-apoptotic proteins BAD and caspase-9, and transcriptional inactivation of FOXO forkhead transcription factors (152). In MKN-45 cells, blockade of CCK2R and COX-2 by AG-041R (a CCK2R antagonist) and NS-398 (a selective COX-2 inhibitor), respectively, was shown to synergistically induce apoptosis through downregulation of the anti-apoptotic protein BCL-2 and upregulation of the pro-apoptotic Bax (95). Similarly, gastrin-induced MCL-1 expression through CCK2R/PKC/MAPK pathway was also shown to be responsible for decreased apoptosis of AGS-CCK2R cells (153). *In vitro* study in human retinal pigment epithelial cells showed that CCK suppresses peroxynitrite-triggered cell apoptosis through inhibiting the expression of Fas, Fas-associated death domain (FADD), caspase-8, and BAX (154). In addition, the level of plasminogen activator inhibitor type 2 (PAI-2), a major gastrin-targeted gene implicated as an inhibitor for cell invasion and apoptosis (155, 156), was shown to be elevated in serum and stomach of hypergastrinemic patients and gastrin-treated media (157). Furthermore, by transfecting AGS-CCK2R cells with PAI-2 promoter-luciferase construct, Dockray and his colleagues showed that gastrin dose- and time-dependently induces the expression of PAI-2 luciferase, likely through Gq/PKC/RhoA-dependent activation of transcription factors CREB and AP1 (157).

Despite the anti-apoptotic effects of gastrin and CCK discussed above, gastrin and CCK were also reported to stimulate apoptosis of various cell lines. In human colorectal cancer Lovo cells expressing endogenous CCK2R and Colo320 cells transfected with WT CCK2R (Colo320wt), 10 nM of gastrin significantly increases the number of apoptotic Lovo cells and Colo320 cells by 21 and 42%, respectively, which is completely abolished in the presence of 500 nM of CCK2R antagonist L365-260 (158). Further *In vitro* signal transduction studies showed that, in Colo320wt cells but not in Colo320 cells lacking CCK2R or expressing loss-of-function CCK2R mutant, gastrin stimulates apoptosis, induces MAPK/ERK/AP-1 cascade, and suppresses the activity of NF-κB, indicating CCK2R mediates gastrin-induced apoptosis (158). Similarly, gastrin-induced apoptosis was also demonstrated in gastric epithelial RGM-1 cells and cholangiocarcinoma Mz-ChA-1 cells (159, 160).

### Gastrin and CCK Effects *in vivo*

To investigate the *in vivo* effects of gastrin on apoptosis, several hypergastrinemic rodent models, such as INS-GAS mice expressing human gastrin minigene spliced with insulin promoter in pancreatic islets, *Mastomys* rodents treated with an H_2_ histamine receptor antagonist, and FVB/N mice treated with a PPI, have been utilized (160–162). Although the ambivalent actions of gastrin were reported in a number of *in vitro* studies, the majority of evidence from *in vivo* studies suggested gastrin signaling through CCK2R stimulates gastric cell apoptosis.

Moss and colleagues, using *TUNEL* technique, showed that *Mastomys* rodents treated with loxtidine, an irreversible H_2_ receptor antagonist, for 8 weeks have a 1.8-fold increase in the apoptotic cells in the hyperplastic mucosa and apoptotic cells return to the control levels upon loxtidine withdraw in 10 days (161). Nevertheless, the ratio of fundic mucosal proliferative to apoptotic cells also increases in the loxtidine-treated *Mastomys* rodents compared to that of the controls (161). In addition, INS-GAS mice and gastrin-infused GAS-KO mice have significantly elevated apoptotic glandular parietal cells, extraglandular mesenchymal cells and infiltrating immune cells, along with increased expression of proapoptotic BAX and decreased expression of BCL-2 compared to the corresponding controls (160). Sustained *H. felis* infection of INS-GAS mice results in exacerbated hypergastrinemia, increased apoptosis, as well as accelerated progression to atrophy (160). Treatment of *H. felis*-infected INS-GAS mice with YF476 (a highly specific CCK2R antagonist) and/or loxtidine for 6 months demonstrated that both agents have equivalent suppressing effects on gastric apoptosis and atrophy, and combination of both drugs exert more profound inhibitory effects on gastric cell apoptosis (160). Using hypergastrinemic mice models including INS-GAS and FVB/N mice treated with omeprazole, Przemeck et al. showed that hypergastrinemia renders gastric epithelial cells more susceptible to induction of apoptosis by 12Gy γ-radiation or *H. pylori* infection, and in both cases these effects are suppressed by CCK2R antagonist YM022 (162).

Similar results were observed in gastric corpus biopsies obtained from *H. pylori*-infected humans with moderate hypergastrinemia (162). One possible mechanism by which gastrin-induced apoptosis results in gastrointestinal cancers was proposed by Houghton et al., through establishment of the *Helicobacter felis*/C57BL/6 mouse model of gastric cancer, who demonstrated that chronic *Helicobacter felis*-induced hypergastrinemia stimulates the apoptosis of gastric stem cells, followed by recruitment and repopulation of bone marrow–derived cells in the gastric mucosa. Subsequently, the bone marrow–derived cells progress through metaplasia and dysplasia to intraepithelial cancer since they are more susceptible to development of malignancy than the originally inhabited gastric epithelial stem cells (163).

In contrast, CCK has been shown to suppress neuronal apoptosis in several animal models. The pilot study of Sugaya et al. demonstrated the ability of CCK to protect the degeneration of cholinergic neurons in a basal forebrain-lesioned rat model, as evidenced by preserved choline acetyltransferase activity and acetylcholine release (164). Similarly, in cultured rat cortical neurons, CCK was shown to inhibit glutamate-induced neuronal death in a dose-dependent manner at concentrations of 1–100 nM. Furthermore, CCK2R was suggested to mediate CCK-induced neuronal protection since this effect was antagonized by the CCK2R antagonist L-365260 but not by the CCK1R antagonist L-364718 (165). In addition, Reisi et al. showed that intraperitoneal injection of CCK-8S, the octapeptide that can rapidly cross the blood-brain barrier and spread across the brain, into male Wistar rats inhibits neuronal apoptosis in the hippocampus, as evidenced by reduced number of TUNEL-positive cells in granular layer of hippocampal dentate gyrus (146). Interestingly. the pilot studies of Lavine et al. demonstrated that CCK is up-regulated and expressed in the pancreatic islet β-cells of obese mice, and whole-body deletion of *Cck* results in reduced islet size and β-cell mass through increased β-cell apoptosis (166). In cultured β-cells or isolated islets, CCK also functions as a paracrine or autocrine factor to protect β-cells from cytokine- or ER stress-stimulating agent-induced apoptosis (166). Similar anti-apoptotic effects of islet-derived CCK were observed in lean transgenic mice that endogenously express CCK in the β-cells (167).

### Mechanism of Gastrin- and CCK-Mediated Effects in Apoptosis

Due to lack of evidence, the exact roles of gastrin and CCK in the regulation of apoptosis are still of debate. Some *in vitro* studies demonstrated that gastrin and CCK exert an anti-apoptotic effect through PI3K/AKT- and p38/AKT-dependent activation of BAD and caspase-8, CCK2R/COX-2-dependent and Fas/FADD/caspase-8-dependent upregulation of BCL-2 and downregulation of BAX, CCK2R/PKC/MAPK-dependent upregulation of MCL-1, as well as PKC/RhoA/CREB/AP1-dependent activation of PAI-2. However, other studies showed gastrin induces apoptosis via activation of MAPK/ERK/AP-1, blockade of NF-κB, and Ca^2+^-dependent activation of PKC-α. Recent *in vivo* studies showed that CCK protects central neurons and pancreatic β-cells from apoptosis in autocrine and/or paracrine manners, whereas the underling mechanisms remain elusive. Further investigations are needed to elucidate gastrin- and CCK-induced effects in apoptosis in the context of cell types and animal models.

## Imaging and Therapeutic Perspectives

It has been well-recognized that both the expression of CCK2R and CCK1R are increased in numerous human NETs over the corresponding normal tissues, suggesting that both receptors might be utilized as molecular targets for localization of certain adenocarcinomas by radiopeptide imaging *in vivo*, and more recently, for treatment by peptide receptor radiation therapy (168, 169). Indeed, inspired by somatostatin receptor scintigraphy, the diagnostic gold standard procedure for the detection of several tumor entities, Gotthardt et al. developed a novel imaging method, gastrin receptor scintigraphy (GRS), for the detection of metastases of medullary thyroid carcinoma (MTC) (170). By comparing different detection methods in 26 patients with metastasized MTC, it was shown that GRS combined with computed tomography is the most effective in MTC detection, with a tumor detection rate of 96.7% (170). In a 60-patient cohort of carcinoids and other NETs the same group suggested that GRS should be performed in selected patients since it may provide additional information in NET patients with equivocal or absent somatostatin uptake (171).

In this scenario, the search of radiolabeled CCK2R ligands with good tumor-to-kidney pharmacodynamics is of great importance in clinical settings. Thirty-four radiolabeled candidate compounds derived from gastrin were screened by measuring tumor and kidney uptake in several pancreatic xenograft nude mouse models, and the peptide with sequence DOTA-HHEAYGWMDF-NH_2_ showed the highest tumor-to-kidney ratio with saturable uptake in target organs and low uptake by non-target tissues, indicating a promising candidate for peptide receptor radiation therapy (172).

Accumulating evidence showed that gastrin signaling via CCK2R stimulates the growth of gastrointestinal cancer cells *in vitro* and *in vivo*, indicating blockade of CCK2R pathway might present a promising strategy for the treatment of gastrointestinal carcinoma. Indeed, in xenografted nude mice transplanted with the mouse colon adenocarcinoma cell line MC-26, proglumide, a weak CCK2R inhibitor, suppresses growth of MC-26 colon cancer and prolongs survival in tumor-bearing mice (173). In addition, decreased mean tumor area, mean tumor weight, and tumor DNA and RNA contents were also observed in proglumide-treated group compared to the control group (173). However, the beneficial effects of proglumide on survival from gastric carcinoma were abolished in a randomized, controlled study of proglumide in 110 gastric tumor patients (174). The authors proposed that more specific and potent CCK2R antagonists, in combination with agents that block gastrin secretion such as somatostatin analogs or prostaglandin analogs, might exert a greater benefit on survival in humans (174).

Furthermore, Watson et al. developed a gastrin-specific monoclonal antibody G17DT, an immunogen composed of the amino terminal portion of G17 linked to a diphtheria toxoid (175). The colon tumor-xenografted rats treated with G17DT were shown to have significantly reduced median cross-sectional tumor area and weights, and increased degree of necrosis compared to control rats (175). Similar survival-promoting effects were observed in severe combined immune deficient mice xenografted with two human gastric cancer lines MGLVA1 cells and ST16 cells (176). In the MGLVA1asc-xenografted mice, the enhancement in survival induced by G17DT was not significantly different from that achieved by treatment with 5-fluorouracil/leucovorin (177). Mice treated with a combination therapy with G17DT and 5-fluorouracil/leucovorin benefit more in survival compared to those treated with G17DT or 5-fluorouracil/leucovorin alone, indicating additive effects of both treatments (177).

Due to the positive results of G17DT achieved in multiple gastrin-sensitive tumor models, the same group further investigated the therapeutic effectiveness of G17DT in clinical trials. G17DT was shown to elicit functional antibodies against gastrin with safe and well-tolerated profile in 52 patients with gastric carcinomas, with the exception that two patients suffered significant adverse reactions (178). In another open-label, multinational, and multicenter phase II study, sixty-five of 94 advanced gastric cancer patients were successfully vaccinated with G17DT in terms of anti-gastrin antibody production and showed longer time-to-progression and median survival compared to control patients (179). A further international multicenter randomized controlled Phase III clinical trial consisting of 154 patients (79 G17DT and 75 placebo) with advanced pancreatic cancer confirmed improved survival of patients in the G17DT group through an intention-to-treat analysis (180). Therefore, G17DT represents a promising therapeutic option for gastrointestinal malignancy.

## Conclusions

Since the discovery of gastrin and CCK a century ago as digestion-related gastrointestinal peptides, our understanding of these peptides have considerably improved. Extensive investigations have demonstrated the expression of gastrin, CCK, and their receptors in a variety of tumor cells and tissues and their involvement in the regulation of cell proliferation and apoptosis, as well as the pathogenesis of cancer. Indeed, a complex network of signaling pathways, including Gq/PLC/Ca^2+^/PKC, Gs/AC/cAMP, and NO/cGMP cascades, activation of kinases such as MAPK, PI3K/AKT, Src, and FAKs, as well as transactivation of the EGFR, have been suggested to contribute to the trophic effects of these two peptides. Finally, diagnosis and treatment approaches targeting peptides and CCK2R, such as receptor scintigraphy and radiopharmaceuticals, have been utilized in tumor imaging and/or therapy *in vitro, in vivo*, and in clinical trials. However, it should be noted that the CCK2R- and CCK1R-mediated signal transduction varies in the context of cell types, suggesting that cautions should be taken in future investigations attempting to target the gastrin and CCK system for the treatment of certain types of cancer.

## Author Contributions

All authors listed have made a substantial, direct and intellectual contribution to the work, and approved it for publication.

### Conflict of Interest

WW and D-YG are employees of Xiamen Huli Guoyu Clinic, Co., Ltd., Xiamen, China. The remaining authors declare that the research was conducted in the absence of any commercial or financial relationships that could be construed as a potential conflict of interest.

## Abbreviations

CCK: cholecystokinin
CCK1R: CCK1 receptor
CCK2R: CCK2 receptor
COX-2: cyclooxygenase-2; epidermal growth factor
EGFR: epidermal growth factor receptor
FAK: focal adhesion tyrosine kinase
Gamide: amidated gastrin
Ggly: Gly-extended gastrin
GRS: gastrin receptor scintigraphy
*H. pylori*: *Helicobacter pylori*
JAK: Janus kinase
MAPK: mitogen-activated protein kinase
MEN-1: multiple endocrine neoplasia type I
MTC: medullary thyroid carcinoma
NETs: neuroendocrine tumors
NO: nitric oxide
ODC: ornithine decarboxylase
PAI-2: plasminogen activator inhibitor type 2
PKB: protein kinase B
PKC: protein kinase C
PPI: proton pump inhibitor
TUNEL: terminal deoxynucleotidyl transferase-mediated dUTP-FITC nick end labeling
WT: wild-type.
